# Diethyl 4-[5-(biphenyl-4-yl)-1*H*-pyrazol-4-yl]-2,6-dimethyl-1,4-dihydro­pyridine-3,5-dicarboxyl­ate ethanol monosolvate

**DOI:** 10.1107/S160053681102349X

**Published:** 2011-06-22

**Authors:** Hoong-Kun Fun, Madhukar Hemamalini, A. M. Vijesh, Arun M. Isloor, T. Arulmoli

**Affiliations:** a X-ray Crystallography Unit, School of Physics, Universiti Sains Malaysia, 11800 USM, Penang, Malaysia; b Department of Chemistry, National Institute of Technology, Karnataka, Surathkal, Mangalore 575 025, India; c SeQuent Scientific Ltd, No. 120 A & B, Industrial Area, Baikampady, New Mangalore, Karnataka 575 011, India

## Abstract

In the title compound, C_28_H_29_N_3_O_4_·C_2_H_6_O, the benzene ring makes dihedral angles of 33.72 (13) and 32.86 (13)°, respectively, with the adjacent pyrazole and phenyl rings. In the crystal, the components are connected *via* inter­molecular N—H⋯O, N—H⋯N, O—H⋯O and C—H⋯O hydrogen bonds, forming a layer parallel to the *bc* plane.

## Related literature

For applications of Hantzsch 1,4-dihydro­pyridines, see: Surendra Kumar *et al.* (2011 ▶); Swarnalatha *et al.* (2011 ▶); Tasaka *et al.* (2001 ▶). For bond-length data, see: Allen *et al.* (1987 ▶).
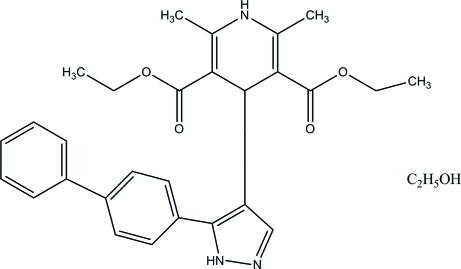


## Experimental

### 

#### Crystal data

C_28_H_29_N_3_O_4_·C_2_H_6_O
*M* *_r_* = 517.61
Orthorhombic, 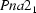

*a* = 34.884 (2) Å
*b* = 10.2322 (7) Å
*c* = 7.8449 (6) Å
*V* = 2800.1 (3) Å^3^
*Z* = 4
Mo *K*α radiation
μ = 0.08 mm^−1^
*T* = 296 K
0.74 × 0.23 × 0.23 mm

#### Data collection

Bruker APEXII DUO CCD area-detector diffractometer
Absorption correction: multi-scan (*SADABS*; Bruker, 2009 ▶) *T*_min_ = 0.941, *T*_max_ = 0.981
19567 measured reflections
4972 independent reflections
4032 reflections with *I* > 2σ(*I*)
*R*_int_ = 0.031

#### Refinement

*R*[*F* ^2^ > 2σ(*F* ^2^)] = 0.045
*wR*(*F* ^2^) = 0.127
*S* = 1.04
4972 reflections
343 parameters
1 restraint
H-atom parameters constrained
Δρ_max_ = 0.22 e Å^−3^
Δρ_min_ = −0.20 e Å^−3^

### 

Data collection: *APEX2* (Bruker, 2009 ▶); cell refinement: *SAINT* (Bruker, 2009 ▶); data reduction: *SAINT*; program(s) used to solve structure: *SHELXTL* (Sheldrick, 2008 ▶); program(s) used to refine structure: *SHELXTL*; molecular graphics: *SHELXTL*; software used to prepare material for publication: *SHELXTL* and *PLATON* (Spek, 2009 ▶).

## Supplementary Material

Crystal structure: contains datablock(s) global, I. DOI: 10.1107/S160053681102349X/is2733sup1.cif

Structure factors: contains datablock(s) I. DOI: 10.1107/S160053681102349X/is2733Isup2.hkl

Supplementary material file. DOI: 10.1107/S160053681102349X/is2733Isup3.cml

Additional supplementary materials:  crystallographic information; 3D view; checkCIF report

## Figures and Tables

**Table 1:** Hydrogen-bond geometry (Å, °)

*D*—H⋯*A*	*D*—H	H⋯*A*	*D*⋯*A*	*D*—H⋯*A*
N1—H1*N*1⋯O5	0.83	2.09	2.880 (3)	158
N3—H1*N*3⋯N2^i^	0.92	2.10	2.958 (2)	155
O5—H1*O*5⋯O1^ii^	0.91	1.88	2.776 (3)	172
C11—H11*A*⋯O2	0.93	2.50	3.414 (2)	167
C25—H25*A*⋯O3	0.96	2.13	2.864 (4)	132

Symmetry codes: (i) 
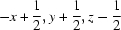
; (ii) 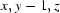.

**Fig. 1.**
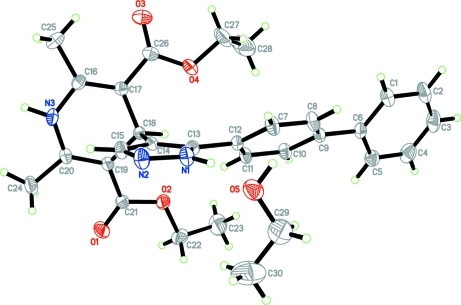
The asymmetric unit of the title compound, showing 30% probability displacement ellipsoids and the atom-numbering scheme.

**Fig. 2.**
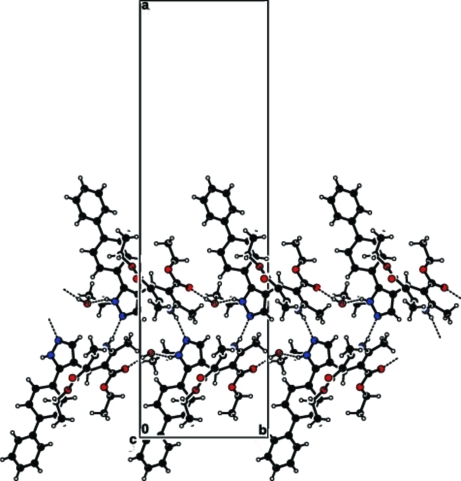
The crystal packing of the title compound.

## supplementary crystallographic information

### Comment

Hantzsch 1,4-dihydropyridines (1,4-DHPs) and their derivatives are an important class of bioactive molecules in the pharmaceutical field. They possess anti-inflammatory, anti-microbial (Surendra Kumar *et al.*, 2011), anti-oxidant and antiulcer activities (Swarnalatha *et al.*, 2011). DHPs are commercially used as calcium channel blockers for the treatment of cardiovascular diseases, including hypertension. Recently, the syntheses of DHPs with respect to Multidrug Resistance (MDR) reversal in tumor cell gave a new dimension to their applications (Tasaka *et al.*, 2001). Keeping in view of the biological importance of 1,4-dihydropyridines, we hereby report the crystal structure of the title compound.

The asymmetric unit of the title compound is shown in Fig. 1. The rings A (N3/C16–C20), B (N1/N2/C13–C15), C (C7–C12) and D (C1–C6) are essentially planar. The dihedral angle between the best planes of these rings are A/B = 89.23 (11)°, A/C = 59.92 (11)°, A/D = 33.06 (12)°, B/C = 33.72 (13)°, B/D = 66.58 (13)° and C/D = 32.86 (13)°.  The bond lengths (Allen *et al.*, 1987) and angles are normal.

In the crystal packing (Fig. 2), the molecules are connected *via* intermolecular N1—H1N1···O5, N3—H1N3···N2, O5—H1O5···O1, C11—H11A···O2 and C25—H25A···O3 (Table 1) hydrogen bonds, forming sheets lying parallel to the *bc*-plane.

### Experimental

3-(4-Biphenyl)-1*H*-pyrazole-4-carbaldehyde (0.2g, 0.80 mmol), ethylacetoacetate (0.21g, 1.6 mmol) and ammonium acetate (0.07g, 0.90 mmol) in ethanol (20 ml) were refluxed for 8 hours in an oil bath. After the completion of the reaction, the reaction mixture was concentrated and then poured onto crushed ice. The precipitated product was filtered and washed with water. The resulting solid was recrystallized from hot ethanol (0.28 g, 74%). M.p. 465–467 K.

### Refinement

All hydrogen atoms were positioned geometrically (N—H = 0.92 or 0.83 Å, O—H = 0.906 Å and C—H = 0.93 or 0.96 Å) and were refined using a riding model, with *U*_iso_(H) = 1.2 or 1.5*U*_eq_(parent atom). A rotating group model was used for the methyl group.  In the absence of significant anomalous scattering effects, 3710 Friedel pairs were merged.

### Crystal data

**Table:**

C_28_H_29_N_3_O_4_·C_2_H_6_O	*F*(000) = 1104
*M**_r_* = 517.61	*D*_x_ = 1.228 Mg m^−^^3^
Orthorhombic, *P**n**a*2_1_	Mo *K*α radiation, λ = 0.71073 Å
Hall symbol: P 2c -2n	Cell parameters from 4868 reflections
*a* = 34.884 (2) Å	θ = 2.7–30.4°
*b* = 10.2322 (7) Å	µ = 0.08 mm^−^^1^
*c* = 7.8449 (6) Å	*T* = 296 K
*V* = 2800.1 (3) Å^3^	Block, colourless
*Z* = 4	0.74 × 0.23 × 0.23 mm

### Data collection

**Table:**

Bruker APEXII DUO CCD area-detector diffractometer	4972 independent reflections
Radiation source: fine-focus sealed tube	4032 reflections with *I* > 2σ(*I*)
graphite	*R*_int_ = 0.031
φ and ω scans	θ_max_ = 31.5°, θ_min_ = 2.7°
Absorption correction: multi-scan (*SADABS*; Bruker, 2009)	*h* = −51→48
*T*_min_ = 0.941, *T*_max_ = 0.981	*k* = −15→15
19567 measured reflections	*l* = −11→11

### Refinement

**Table:**

Refinement on *F*^2^	Primary atom site location: structure-invariant direct methods
Least-squares matrix: full	Secondary atom site location: difference Fourier map
*R*[*F*^2^ > 2σ(*F*^2^)] = 0.045	Hydrogen site location: inferred from neighbouring sites
*wR*(*F*^2^) = 0.127	H-atom parameters constrained
*S* = 1.04	*w* = 1/[σ^2^(*F*_o_^2^) + (0.0683*P*)^2^ + 0.2544*P*] where *P* = (*F*_o_^2^ + 2*F*_c_^2^)/3
4972 reflections	(Δ/σ)_max_ = 0.001
343 parameters	Δρ_max_ = 0.22 e Å^−^^3^
1 restraint	Δρ_min_ = −0.20 e Å^−^^3^

### Special details

**Table:**

Geometry. All s.u.'s (except the s.u. in the dihedral angle between two l.s. planes) are estimated using the full covariance matrix. The cell s.u.'s are taken into account individually in the estimation of s.u.'s in distances, angles and torsion angles; correlations between s.u.'s in cell parameters are only used when they are defined by crystal symmetry. An approximate (isotropic) treatment of cell s.u.'s is used for estimating s.u.'s involving l.s. planes.
Refinement. Refinement of F^2^ against ALL reflections. The weighted R-factor wR and goodness of fit S are based on F^2^, conventional R-factors R are based on F, with F set to zero for negative F^2^. The threshold expression of F^2^ > 2σ(F^2^) is used only for calculating R-factors(gt) etc. and is not relevant to the choice of reflections for refinement. R-factors based on F^2^ are statistically about twice as large as those based on F, and R- factors based on ALL data will be even larger.

### Fractional atomic coordinates and isotropic or equivalent isotropic displacement parameters (Å^2^)

**Table:**

	*x*	*y*	*z*	*U*_iso_*/*U*_eq_	
O1	0.15693 (5)	0.88345 (16)	0.3346 (3)	0.0591 (5)	
O2	0.11537 (4)	0.72014 (13)	0.3073 (2)	0.0436 (3)	
O3	0.13653 (8)	0.4760 (3)	−0.3954 (3)	0.0946 (9)	
O4	0.10858 (5)	0.43817 (19)	−0.1476 (3)	0.0645 (5)	
N1	0.18843 (4)	0.30435 (15)	0.2742 (3)	0.0389 (4)	
H1N1	0.1834	0.2394	0.3337	0.047*	
N2	0.22190 (4)	0.36422 (16)	0.2390 (3)	0.0441 (4)	
N3	0.20862 (5)	0.73950 (16)	−0.1187 (3)	0.0408 (4)	
H1N3	0.2252	0.7840	−0.1892	0.049*	
C1	−0.00634 (6)	0.0493 (2)	0.2277 (4)	0.0495 (6)	
H1A	0.0112	0.0049	0.1599	0.059*	
C2	−0.04339 (7)	0.0011 (2)	0.2442 (5)	0.0619 (7)	
H2A	−0.0505	−0.0743	0.1861	0.074*	
C3	−0.06951 (7)	0.0647 (3)	0.3461 (5)	0.0664 (8)	
H3A	−0.0943	0.0322	0.3574	0.080*	
C4	−0.05887 (7)	0.1764 (3)	0.4314 (4)	0.0617 (7)	
H4A	−0.0764	0.2194	0.5008	0.074*	
C5	−0.02180 (6)	0.2254 (2)	0.4138 (3)	0.0475 (5)	
H5A	−0.0148	0.3011	0.4718	0.057*	
C6	0.00488 (5)	0.16286 (17)	0.3112 (3)	0.0367 (4)	
C7	0.11209 (5)	0.18553 (17)	0.2385 (4)	0.0423 (5)	
H7A	0.1326	0.1287	0.2219	0.051*	
C8	0.07570 (5)	0.13530 (16)	0.2610 (4)	0.0439 (5)	
H8A	0.0721	0.0453	0.2582	0.053*	
C9	0.04412 (5)	0.21663 (17)	0.2879 (3)	0.0342 (4)	
C10	0.05099 (5)	0.35085 (17)	0.2899 (3)	0.0382 (4)	
H10A	0.0305	0.4077	0.3071	0.046*	
C11	0.08745 (5)	0.40188 (15)	0.2671 (3)	0.0361 (4)	
H11A	0.0910	0.4919	0.2698	0.043*	
C12	0.11871 (4)	0.32017 (15)	0.2402 (3)	0.0302 (3)	
C13	0.15760 (5)	0.37296 (16)	0.2171 (3)	0.0299 (3)	
C14	0.21198 (5)	0.47197 (18)	0.1555 (3)	0.0383 (4)	
H14A	0.2295	0.5323	0.1129	0.046*	
C15	0.17206 (5)	0.48438 (15)	0.1388 (2)	0.0286 (3)	
C16	0.18842 (5)	0.65135 (18)	−0.2170 (3)	0.0356 (4)	
C17	0.15876 (5)	0.58515 (17)	−0.1458 (3)	0.0314 (3)	
C18	0.15176 (4)	0.59474 (15)	0.0457 (2)	0.0272 (3)	
H18A	0.1242	0.5870	0.0666	0.033*	
C19	0.16513 (5)	0.72775 (15)	0.1099 (3)	0.0307 (3)	
C20	0.19517 (5)	0.78817 (17)	0.0321 (3)	0.0366 (4)	
C21	0.14687 (5)	0.78599 (15)	0.2587 (3)	0.0342 (4)	
C22	0.09488 (8)	0.7696 (2)	0.4542 (4)	0.0549 (6)	
H22A	0.0946	0.8644	0.4528	0.066*	
H22B	0.1072	0.7409	0.5585	0.066*	
C23	0.05559 (10)	0.7187 (4)	0.4459 (6)	0.0987 (15)	
H23A	0.0413	0.7498	0.5421	0.148*	
H23B	0.0562	0.6249	0.4474	0.148*	
H23C	0.0436	0.7481	0.3426	0.148*	
C24	0.21576 (7)	0.9085 (2)	0.0939 (4)	0.0575 (7)	
H24A	0.2358	0.9306	0.0151	0.086*	
H24B	0.2267	0.8917	0.2041	0.086*	
H24C	0.1979	0.9797	0.1018	0.086*	
C25	0.20259 (7)	0.6421 (3)	−0.3981 (3)	0.0527 (6)	
H25A	0.1875	0.5793	−0.4593	0.079*	
H25B	0.2290	0.6153	−0.3982	0.079*	
H25C	0.2003	0.7260	−0.4521	0.079*	
C26	0.13454 (6)	0.4967 (2)	−0.2460 (3)	0.0429 (5)	
C27	0.08260 (8)	0.3459 (3)	−0.2227 (5)	0.0736 (9)	
H27A	0.0854	0.2619	−0.1668	0.088*	
H27B	0.0888	0.3347	−0.3424	0.088*	
C28	0.04346 (9)	0.3905 (4)	−0.2063 (7)	0.1010 (14)	
H28A	0.0265	0.3277	−0.2571	0.151*	
H28B	0.0406	0.4730	−0.2631	0.151*	
H28C	0.0372	0.4004	−0.0878	0.151*	
O5	0.19324 (6)	0.08630 (16)	0.5054 (3)	0.0593 (5)	
H1O5	0.1832	0.0202	0.4429	0.089*	
C29	0.17458 (13)	0.0994 (4)	0.6678 (6)	0.0951 (12)	
H29A	0.1753	0.0167	0.7281	0.114*	
H29B	0.1479	0.1234	0.6512	0.114*	
C30	0.19408 (15)	0.2004 (4)	0.7688 (7)	0.1167 (16)	
H30A	0.1815	0.2095	0.8769	0.175*	
H30B	0.1932	0.2821	0.7088	0.175*	
H30C	0.2203	0.1755	0.7865	0.175*	

### Atomic displacement parameters (Å^2^)

**Table:**

	*U*^11^	*U*^22^	*U*^33^	*U*^12^	*U*^13^	*U*^23^
O1	0.0662 (10)	0.0450 (8)	0.0661 (12)	−0.0165 (7)	0.0147 (10)	−0.0243 (9)
O2	0.0459 (7)	0.0403 (6)	0.0446 (9)	−0.0068 (5)	0.0161 (7)	−0.0116 (7)
O3	0.1151 (19)	0.129 (2)	0.0396 (10)	−0.0564 (16)	0.0000 (12)	−0.0216 (13)
O4	0.0597 (10)	0.0770 (11)	0.0569 (11)	−0.0369 (9)	0.0027 (9)	−0.0219 (10)
N1	0.0317 (7)	0.0343 (7)	0.0507 (10)	−0.0001 (5)	−0.0060 (8)	0.0109 (8)
N2	0.0274 (7)	0.0448 (8)	0.0601 (12)	0.0007 (6)	−0.0083 (8)	0.0113 (9)
N3	0.0319 (7)	0.0415 (8)	0.0490 (11)	−0.0074 (6)	0.0100 (8)	0.0044 (8)
C1	0.0445 (10)	0.0399 (9)	0.0641 (16)	−0.0092 (8)	−0.0029 (11)	0.0032 (11)
C2	0.0519 (12)	0.0499 (11)	0.084 (2)	−0.0227 (9)	−0.0094 (15)	0.0067 (14)
C3	0.0393 (11)	0.0753 (16)	0.085 (2)	−0.0209 (11)	−0.0035 (14)	0.0224 (17)
C4	0.0380 (11)	0.0818 (17)	0.0655 (17)	−0.0045 (11)	0.0085 (12)	0.0111 (16)
C5	0.0389 (10)	0.0542 (11)	0.0493 (13)	−0.0060 (8)	0.0020 (10)	0.0023 (11)
C6	0.0322 (8)	0.0355 (8)	0.0423 (11)	−0.0057 (6)	−0.0024 (8)	0.0095 (8)
C7	0.0317 (8)	0.0285 (7)	0.0668 (15)	0.0015 (6)	−0.0008 (10)	0.0024 (9)
C8	0.0358 (8)	0.0268 (7)	0.0690 (15)	−0.0035 (6)	−0.0028 (10)	0.0056 (9)
C9	0.0321 (8)	0.0335 (7)	0.0370 (10)	−0.0059 (6)	−0.0027 (8)	0.0036 (8)
C10	0.0304 (7)	0.0314 (7)	0.0529 (12)	−0.0013 (6)	0.0050 (9)	−0.0024 (9)
C11	0.0340 (8)	0.0264 (6)	0.0480 (11)	−0.0030 (6)	0.0051 (9)	−0.0016 (8)
C12	0.0278 (7)	0.0289 (6)	0.0339 (9)	−0.0031 (5)	−0.0005 (7)	0.0035 (7)
C13	0.0279 (7)	0.0288 (7)	0.0331 (9)	−0.0004 (5)	−0.0035 (7)	0.0009 (7)
C14	0.0276 (8)	0.0397 (8)	0.0475 (12)	−0.0036 (6)	−0.0046 (8)	0.0064 (9)
C15	0.0265 (7)	0.0282 (6)	0.0311 (8)	−0.0012 (6)	−0.0022 (7)	−0.0006 (7)
C16	0.0339 (8)	0.0380 (8)	0.0349 (9)	0.0051 (6)	0.0042 (8)	0.0043 (8)
C17	0.0291 (7)	0.0333 (7)	0.0319 (9)	0.0025 (6)	−0.0027 (7)	0.0006 (7)
C18	0.0236 (6)	0.0278 (6)	0.0301 (8)	0.0000 (5)	0.0002 (6)	0.0002 (7)
C19	0.0286 (7)	0.0276 (6)	0.0360 (9)	−0.0016 (6)	0.0006 (7)	−0.0008 (7)
C20	0.0312 (8)	0.0313 (7)	0.0472 (12)	−0.0044 (6)	0.0008 (8)	0.0004 (8)
C21	0.0381 (8)	0.0281 (7)	0.0363 (10)	−0.0002 (6)	0.0004 (8)	−0.0017 (8)
C22	0.0671 (15)	0.0489 (11)	0.0486 (13)	0.0036 (10)	0.0219 (13)	−0.0101 (11)
C23	0.077 (2)	0.105 (2)	0.114 (3)	−0.0266 (18)	0.060 (2)	−0.054 (3)
C24	0.0530 (12)	0.0465 (11)	0.0730 (18)	−0.0226 (9)	0.0106 (13)	−0.0085 (12)
C25	0.0559 (13)	0.0648 (13)	0.0374 (11)	0.0067 (11)	0.0145 (11)	0.0054 (11)
C26	0.0450 (10)	0.0459 (10)	0.0378 (11)	−0.0010 (8)	−0.0067 (9)	−0.0050 (9)
C27	0.0620 (15)	0.0731 (16)	0.086 (2)	−0.0284 (13)	−0.0082 (17)	−0.0258 (18)
C28	0.0580 (17)	0.131 (3)	0.114 (4)	−0.0168 (18)	−0.015 (2)	−0.036 (3)
O5	0.0741 (11)	0.0425 (8)	0.0612 (12)	−0.0067 (7)	−0.0091 (10)	0.0011 (8)
C29	0.108 (3)	0.088 (2)	0.089 (3)	−0.013 (2)	0.011 (3)	−0.008 (2)
C30	0.164 (4)	0.098 (3)	0.088 (3)	0.031 (3)	−0.012 (3)	−0.023 (3)

### Geometric parameters (Å, °)

**Table:**

O1—C21	1.213 (2)	C14—H14A	0.9300
O2—C21	1.344 (2)	C15—C18	1.520 (2)
O2—C22	1.448 (3)	C16—C17	1.357 (3)
O3—C26	1.193 (3)	C16—C25	1.507 (3)
O4—C26	1.332 (3)	C17—C26	1.466 (3)
O4—C27	1.435 (3)	C17—C18	1.525 (3)
N1—N2	1.347 (2)	C18—C19	1.524 (2)
N1—C13	1.360 (2)	C18—H18A	0.9800
N1—H1N1	0.8303	C19—C20	1.361 (2)
N2—C14	1.328 (3)	C19—C21	1.457 (3)
N3—C20	1.367 (3)	C20—C24	1.505 (3)
N3—C16	1.380 (3)	C22—C23	1.468 (4)
N3—H1N3	0.9208	C22—H22A	0.9700
C1—C2	1.389 (3)	C22—H22B	0.9700
C1—C6	1.390 (3)	C23—H23A	0.9600
C1—H1A	0.9300	C23—H23B	0.9600
C2—C3	1.376 (5)	C23—H23C	0.9600
C2—H2A	0.9300	C24—H24A	0.9600
C3—C4	1.376 (4)	C24—H24B	0.9600
C3—H3A	0.9300	C24—H24C	0.9600
C4—C5	1.394 (3)	C25—H25A	0.9600
C4—H4A	0.9300	C25—H25B	0.9600
C5—C6	1.387 (3)	C25—H25C	0.9600
C5—H5A	0.9300	C27—C28	1.446 (5)
C6—C9	1.487 (2)	C27—H27A	0.9700
C7—C8	1.381 (2)	C27—H27B	0.9700
C7—C12	1.397 (2)	C28—H28A	0.9600
C7—H7A	0.9300	C28—H28B	0.9600
C8—C9	1.397 (3)	C28—H28C	0.9600
C8—H8A	0.9300	O5—C29	1.437 (5)
C9—C10	1.394 (2)	O5—H1O5	0.9060
C10—C11	1.387 (2)	C29—C30	1.469 (6)
C10—H10A	0.9300	C29—H29A	0.9700
C11—C12	1.390 (2)	C29—H29B	0.9700
C11—H11A	0.9300	C30—H30A	0.9600
C12—C13	1.471 (2)	C30—H30B	0.9600
C13—C15	1.390 (2)	C30—H30C	0.9600
C14—C15	1.405 (2)		
			
C21—O2—C22	117.03 (17)	C19—C18—H18A	108.5
C26—O4—C27	119.2 (2)	C17—C18—H18A	108.5
N2—N1—C13	112.53 (15)	C20—C19—C21	120.64 (16)
N2—N1—H1N1	131.3	C20—C19—C18	119.56 (17)
C13—N1—H1N1	115.6	C21—C19—C18	119.73 (15)
C14—N2—N1	104.64 (14)	C19—C20—N3	119.11 (17)
C20—N3—C16	123.13 (16)	C19—C20—C24	126.5 (2)
C20—N3—H1N3	123.7	N3—C20—C24	114.38 (19)
C16—N3—H1N3	108.0	O1—C21—O2	120.59 (19)
C2—C1—C6	121.0 (2)	O1—C21—C19	127.07 (18)
C2—C1—H1A	119.5	O2—C21—C19	112.33 (15)
C6—C1—H1A	119.5	O2—C22—C23	107.5 (2)
C3—C2—C1	120.1 (2)	O2—C22—H22A	110.2
C3—C2—H2A	119.9	C23—C22—H22A	110.2
C1—C2—H2A	119.9	O2—C22—H22B	110.2
C4—C3—C2	119.8 (2)	C23—C22—H22B	110.2
C4—C3—H3A	120.1	H22A—C22—H22B	108.5
C2—C3—H3A	120.1	C22—C23—H23A	109.5
C3—C4—C5	120.1 (3)	C22—C23—H23B	109.5
C3—C4—H4A	120.0	H23A—C23—H23B	109.5
C5—C4—H4A	120.0	C22—C23—H23C	109.5
C6—C5—C4	120.9 (2)	H23A—C23—H23C	109.5
C6—C5—H5A	119.5	H23B—C23—H23C	109.5
C4—C5—H5A	119.5	C20—C24—H24A	109.5
C5—C6—C1	118.04 (18)	C20—C24—H24B	109.5
C5—C6—C9	121.22 (18)	H24A—C24—H24B	109.5
C1—C6—C9	120.73 (19)	C20—C24—H24C	109.5
C8—C7—C12	121.19 (16)	H24A—C24—H24C	109.5
C8—C7—H7A	119.4	H24B—C24—H24C	109.5
C12—C7—H7A	119.4	C16—C25—H25A	109.5
C7—C8—C9	121.51 (15)	C16—C25—H25B	109.5
C7—C8—H8A	119.2	H25A—C25—H25B	109.5
C9—C8—H8A	119.2	C16—C25—H25C	109.5
C10—C9—C8	116.95 (15)	H25A—C25—H25C	109.5
C10—C9—C6	121.42 (16)	H25B—C25—H25C	109.5
C8—C9—C6	121.62 (16)	O3—C26—O4	121.9 (2)
C11—C10—C9	121.81 (16)	O3—C26—C17	127.1 (2)
C11—C10—H10A	119.1	O4—C26—C17	111.0 (2)
C9—C10—H10A	119.1	O4—C27—C28	110.6 (3)
C10—C11—C12	120.84 (15)	O4—C27—H27A	109.5
C10—C11—H11A	119.6	C28—C27—H27A	109.5
C12—C11—H11A	119.6	O4—C27—H27B	109.5
C11—C12—C7	117.70 (15)	C28—C27—H27B	109.5
C11—C12—C13	121.41 (14)	H27A—C27—H27B	108.1
C7—C12—C13	120.89 (15)	C27—C28—H28A	109.5
N1—C13—C15	106.37 (15)	C27—C28—H28B	109.5
N1—C13—C12	119.93 (15)	H28A—C28—H28B	109.5
C15—C13—C12	133.66 (15)	C27—C28—H28C	109.5
N2—C14—C15	112.28 (16)	H28A—C28—H28C	109.5
N2—C14—H14A	123.9	H28B—C28—H28C	109.5
C15—C14—H14A	123.9	C29—O5—H1O5	112.0
C13—C15—C14	104.16 (15)	O5—C29—C30	109.5 (4)
C13—C15—C18	130.75 (14)	O5—C29—H29A	109.8
C14—C15—C18	125.01 (15)	C30—C29—H29A	109.8
C17—C16—N3	119.08 (19)	O5—C29—H29B	109.8
C17—C16—C25	127.3 (2)	C30—C29—H29B	109.8
N3—C16—C25	113.57 (19)	H29A—C29—H29B	108.2
C16—C17—C26	121.8 (2)	C29—C30—H30A	109.5
C16—C17—C18	119.68 (17)	C29—C30—H30B	109.5
C26—C17—C18	118.38 (17)	H30A—C30—H30B	109.5
C15—C18—C19	111.22 (14)	C29—C30—H30C	109.5
C15—C18—C17	110.55 (14)	H30A—C30—H30C	109.5
C19—C18—C17	109.50 (15)	H30B—C30—H30C	109.5
C15—C18—H18A	108.5		
			
C13—N1—N2—C14	−1.0 (3)	C20—N3—C16—C25	162.70 (19)
C6—C1—C2—C3	−1.0 (4)	N3—C16—C17—C26	175.86 (17)
C1—C2—C3—C4	0.2 (5)	C25—C16—C17—C26	−3.3 (3)
C2—C3—C4—C5	0.3 (5)	N3—C16—C17—C18	−8.4 (3)
C3—C4—C5—C6	−0.1 (4)	C25—C16—C17—C18	172.41 (19)
C4—C5—C6—C1	−0.6 (4)	C13—C15—C18—C19	130.8 (2)
C4—C5—C6—C9	178.2 (2)	C14—C15—C18—C19	−52.8 (2)
C2—C1—C6—C5	1.1 (4)	C13—C15—C18—C17	−107.3 (2)
C2—C1—C6—C9	−177.7 (2)	C14—C15—C18—C17	69.0 (2)
C12—C7—C8—C9	−0.6 (4)	C16—C17—C18—C15	−92.78 (19)
C7—C8—C9—C10	0.4 (4)	C26—C17—C18—C15	83.07 (19)
C7—C8—C9—C6	179.6 (2)	C16—C17—C18—C19	30.1 (2)
C5—C6—C9—C10	−32.4 (3)	C26—C17—C18—C19	−154.05 (15)
C1—C6—C9—C10	146.4 (2)	C15—C18—C19—C20	91.1 (2)
C5—C6—C9—C8	148.4 (2)	C17—C18—C19—C20	−31.4 (2)
C1—C6—C9—C8	−32.8 (3)	C15—C18—C19—C21	−85.96 (19)
C8—C9—C10—C11	−0.2 (4)	C17—C18—C19—C21	151.56 (16)
C6—C9—C10—C11	−179.5 (2)	C21—C19—C20—N3	−172.09 (17)
C9—C10—C11—C12	0.3 (4)	C18—C19—C20—N3	10.9 (3)
C10—C11—C12—C7	−0.5 (3)	C21—C19—C20—C24	6.8 (3)
C10—C11—C12—C13	−179.5 (2)	C18—C19—C20—C24	−170.2 (2)
C8—C7—C12—C11	0.6 (4)	C16—N3—C20—C19	15.3 (3)
C8—C7—C12—C13	179.7 (2)	C16—N3—C20—C24	−163.8 (2)
N2—N1—C13—C15	0.4 (2)	C22—O2—C21—O1	−1.0 (3)
N2—N1—C13—C12	178.27 (19)	C22—O2—C21—C19	−179.95 (19)
C11—C12—C13—N1	147.1 (2)	C20—C19—C21—O1	−5.4 (3)
C7—C12—C13—N1	−31.9 (3)	C18—C19—C21—O1	171.6 (2)
C11—C12—C13—C15	−35.7 (3)	C20—C19—C21—O2	173.48 (18)
C7—C12—C13—C15	145.3 (2)	C18—C19—C21—O2	−9.5 (2)
N1—N2—C14—C15	1.2 (3)	C21—O2—C22—C23	158.7 (3)
N1—C13—C15—C14	0.4 (2)	C27—O4—C26—O3	1.0 (4)
C12—C13—C15—C14	−177.1 (2)	C27—O4—C26—C17	−178.9 (2)
N1—C13—C15—C18	177.32 (19)	C16—C17—C26—O3	−2.9 (4)
C12—C13—C15—C18	−0.2 (4)	C18—C17—C26—O3	−178.7 (3)
N2—C14—C15—C13	−1.0 (2)	C16—C17—C26—O4	176.97 (18)
N2—C14—C15—C18	−178.21 (19)	C18—C17—C26—O4	1.2 (3)
C20—N3—C16—C17	−16.6 (3)	C26—O4—C27—C28	−117.7 (4)

### Hydrogen-bond geometry (Å, °)

**Table:**

*D*—H···*A*	*D*—H	H···*A*	*D*···*A*	*D*—H···*A*
N1—H1N1···O5	0.83	2.09	2.880 (3)	158
N3—H1N3···N2^i^	0.92	2.10	2.958 (2)	155
O5—H1O5···O1^ii^	0.91	1.88	2.776 (3)	172
C11—H11A···O2	0.93	2.50	3.414 (2)	167
C25—H25A···O3	0.96	2.13	2.864 (4)	132

Symmetry codes: (i) −*x*+1/2, *y*+1/2, *z*−1/2; (ii) *x*, *y*−1, *z*.
